# Risk of Cardiac Implantable Electronic Device Infection after Early versus Delayed Lead Repositioning

**DOI:** 10.3390/jcdd11040117

**Published:** 2024-04-09

**Authors:** Noemi Schvartz, Arian Haidary, Reza Wakili, Florian Hecker, Jana Kupusovic, Elod-Janos Zsigmond, Marton Miklos, Laszlo Saghy, Tamas Szili-Torok, Julia W. Erath, Mate Vamos

**Affiliations:** 1 Cardiology Center/Cardiac Electrophysiology Division, Internal Medicine Clinic, University of Szeged, 6725 Szeged, Hungary; noemi.schvartz@gmail.com (N.S.);; 2 Department of Cardiology, Division of Clinical Electrophysiology, Goethe University Hospital Frankfurt, 60596 Frankfurt am Main, Germany; 3 Department of Cardiac Surgery, Goethe University Hospital Frankfurt, 60596 Frankfurt am Main, Germany; 4 Doctoral School of Clinical Medicine, University of Szeged, 6725 Szeged, Hungary; 5 Central Hospital of Northern Pest—Military Hospital, 1134 Budapest, Hungary

**Keywords:** cardiac implantable electronic device, CIED, infection, reoperation, reintervention, lead, dislodgement, extraction, pacemaker, ICD, CRT

## Abstract

(1) Background: Early reintervention increases the risk of infection of cardiac implantable electronic devices (CIEDs). Some operators therefore delay lead repositioning in the case of dislocation by weeks; however, there is no evidence to support this practice. The aim of our study was to evaluate the impact of the timing of reoperation on infection risk. (2) Methods: The data from consecutive patients undergoing lead repositioning in two European referral centers were retrospectively analyzed. The odds ratio (OR) of CIED infection in the first year was compared among patients undergoing early (≤1 week) vs. delayed (>1 week to 1 year) reoperation. (3) Results: Out of 249 patients requiring CIED reintervention, 85 patients (34%) underwent an early (median 2 days) and 164 (66%) underwent a delayed lead revision (median 53 days). A total of nine (3.6%) wound/device infections were identified. The risk of infection was numerically lower in the early (1.2%) vs. delayed (4.9%) intervention group yielding no statistically significant difference, even after adjustment for typical risk factors for CIED infection (adjusted OR = 0.264, 95% CI 0.032–2.179, *p* = 0.216). System explantation/extraction was necessary in seven cases, all being revised in the delayed group. (4) Conclusions: In this bicentric, international study, delayed lead repositioning did not reduce the risk of CIED infection.

## 1. Introduction

Cardiac implantable electronic devices (CIEDs) are basic elements in the treatment of different cardiac arrhythmias and heart failure [1,2,3,4,5]. Although CIEDs have become increasingly safe and effective due to the significant technical advances of the last decades, a substantial risk for complications still persists. CIED infection is deemed to be one of the most serious complications since it requires complete system removal with all possible risks of transvenous lead extraction [6,7,8,9,10,11]. Although many preventive strategies—for instance, administration of prophylactic antibiotic therapy before implantation or antibiotic-eluting envelopes for high-risk patients—have been established and were able to demonstrate lower infection rates, uncertainties still exist about other regimens and protocols [9,12,13,14].

A reoperation involving a pocket opening is one of the important risk factors for device-related infections [15,16,17,18]. Especially, the early reinterventions are associated with an increased risk of CIED infection [18,19]. Accordingly, the 2019 EHRA/HRS consensus document emphasizes that all measures must be taken to avoid this need (i.e., avoid hematoma, lead dislodgment, etc.) [9]. 

Some operators therefore delay lead repositioning in the case of lead dislocation by weeks. However, there is no evidence to support this practice. The aim of our study was to evaluate the impact of the timing of reoperation on infection risk. 

## 2. Methods

### 2.1. Patient Population

The clinical data from consecutive patients undergoing a reintervention for lead dislocation within the first year of the primary CIED implantation at the J. W. Goethe University (Frankfurt, Germany) and the University of Szeged (Szeged, Hungary) were retrospectively analyzed between January 1995 and August 2022. All types of CIED implantations with transvenous leads regardless of the manufacturer were considered (i.e., single- or dual-chamber pacemakers or defibrillators, cardiac resynchronization pacemakers or defibrillators). Patients were included if they underwent a reoperation either for lead dislocation/dysfunction or generator replacement within the first year of the primary implantation. Reoperation for sole evacuation of a pocket hematoma and upgrade procedures, defined as the addition of any further leads, were exclusion criteria.

The patients were divided into two groups based on the timing of the reintervention relative to the implantation. Early revision was defined as a reoperation performed within one week after the primary implantation, while delayed revision meant a reintervention after the first week of the primary operation but not later than one year. The selection of the 1-week cut-off was made pragmatically, taking into account that hospitalization with a lead revision typically does not extend beyond one week. This study was approved by the institutional review boards of the participating centers (J. W. Goethe University: No. 264/18; and University of Szeged: No. 4871) and complies with the ethical guidelines of the Declaration of Helsinki.

### 2.2. Study Endpoints

The clinical outcomes of the current study were a device-related infection and transvenous lead extraction due to infection, both within the first year after the reintervention. CIED infection and transvenous lead extraction (TLE) were defined concordant to the current EHRA consensus documents [9,10].

To assess the impact of different risk factors for CIED infection, known predisposing clinical parameters of infection were also collected, such as number of implanted leads, diabetes, fever prior to the implant, prolonged antibiotic therapy, anticoagulation, antiplatelet therapy, corticosteroid use and temporary pacemaker implantation. Laboratory markers such as the white blood cell count, C-reactive protein and creatinine levels were also collected at the time of the primary implantation and revision, when they were available. 

### 2.3. Statistical Analysis 

A statistical analysis was performed using SPSS Statistics software, version 25.0 (IBM, Armonk, NY, USA). The Kolmogorov–Smirnov test was used to evaluate the normal distribution of continuous data. The χ^2^ test was used to test for categorical variables and the 2-sample *t* test or the Mann–Whitney U test for continuous variables among patients’ groups.

The effect of early vs. delayed reintervention on infection was assessed by the odds ratio (OR) with a 95% confidence interval (CI). To overcome the problem of zero event cells, the Haldane–Anscombe correction was used to calculate the unadjusted OR for the explantation/extraction [MedCalc Software Ltd. Odds ratio calculator; https://www.medcalc.org/calc/odds_ratio.php (Version 22.016; accessed on 20 November 2023)]. The statistical model was also adjusted for the typical risk factors of CIED infection using a binary multivariate logistic regression analysis. Two-sided *p* values < 0.05 were considered statistically significant.

## 3. Results

### 3.1. Patients Characteristics

A total of 249 patients (Frankfurt *N* = 74 and Szeged *N* = 175) were included in this study, of whom 85 (34%) underwent an early and 164 (66%) a delayed reoperation. The reinterventions were performed predominantly due to lead repositioning, except one case in whom the generator should have been replaced after 82 days due to a superficial location. The median time to revision was 2 days (interquartile range (IQR): 1–4.5) in the early and 53 days (IQR: 36–209) in the delayed group.

**Figure 1:**
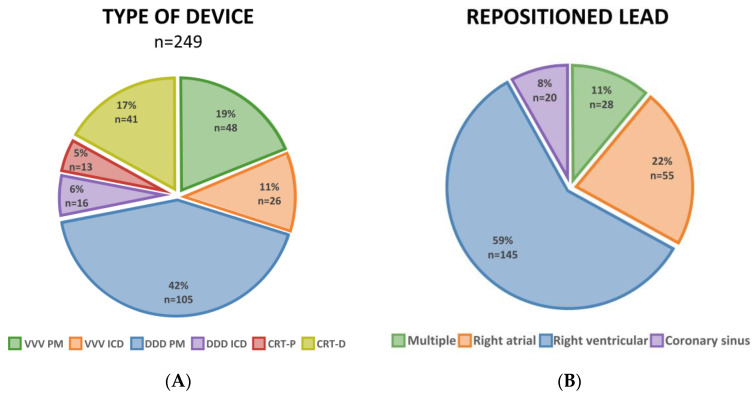
Types of devices requiring reintervention (**A**) and repositioned leads (**B**).

The distributions of the device types and repositioned leads are shown in Figure 1A,B. Reintervention was most frequently required for dual-chamber systems (48% of all cases), and the most frequently repositioned lead was the right ventricular lead (59% of all cases). The patients in the early intervention group were older (76.0 (IQR 68.9–81.8) vs. 69.4 (IQR 62.3–77.9) years, *p* = 0.001) but had fewer implanted leads than the patients with delayed intervention (1.7 ± 0.7 vs. 2.0 ± 0.7, *p* = 0.006). There was no significant difference between the two groups in terms of other comorbidities serving as predisposing factors of infection. The laboratory markers, like the creatinine and baseline C-reactive protein levels, although not available for all the patients, indicated rather an increased risk for infection in the early compared to the delayed patient group. All the baseline characteristics are shown in Table 1. 

### 3.2. Study Endpoints

**Figure 2:**
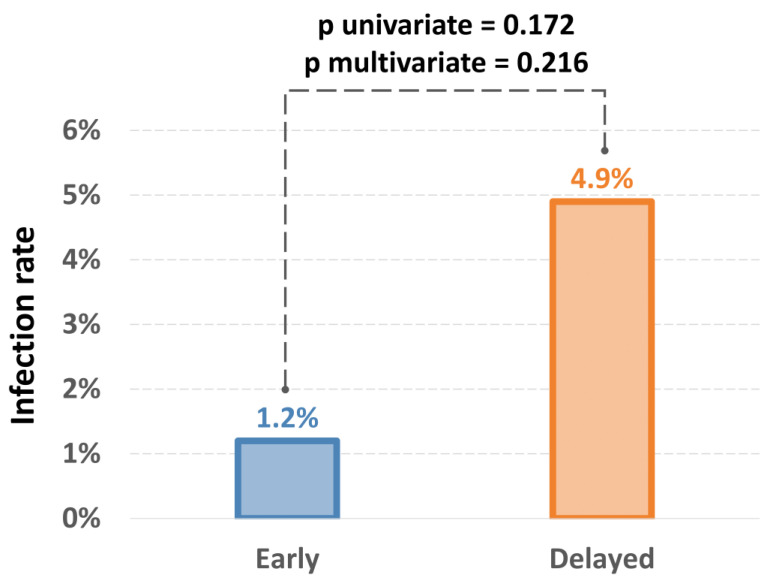
Infections and extractions due to infection in the early versus delayed groups.

A total of nine patients (3.6%) developed a CIED infection, one patient (1.2%) in the early and eight patients (4.9%) in the delayed intervention group (OR = 0.232; 95% CI 0.029–1.888; *p* = 0.172) (Table 2) (Figure 2). After adjustment for typical risk factors for CIED infection (i.e., number of implanted leads, diabetes, chronic heart failure, fever prior to implantation, therapy with corticosteroid, anticoagulation or antiplatelet therapy and temporary pacemaker), this difference remained non-significant (adjusted OR = 0.264, 95% CI 0.032–2.179, *p* = 0.216) (Table 3 and Table 4). Of note, only a fever prior to implantation from the analyzed risk factors proved to be an independent predictor for CIED infection in the current cohort.

Two out of the nine primary outcome event cases had only incisional superficial inflammation that resolved conservatively after treatment with antibiotic therapy. Seven patients (2.8%) required complete system explantation/lead extraction due to infection; these patients were in the delayed intervention group (unadjusted OR 0.128, 95% CI 0.01–2.273, *p* = 0.161).

## 4. Discussion

### 4.1. Main Findings

To the best of our knowledge, our bicentric, international, retrospective study is the first in the literature evaluating the impact of the timing of reintervention for a lead dislocation on infection risk in patients with CIEDs. The incidence of infection was numerically lower in the early (<1 week) versus the delayed intervention group, yielding no statistically significant difference neither in univariate nor in multivariate statistical comparisons. Based on our results, the strategy of delaying lead repositioning in the case of lead dislocation by weeks does not reduce the incidence of device infection. 

### 4.2. Known Risk Factors of CIED Infection

One of the most serious and life-threatening complications associated with transvenous cardiac electronic device implantation is the infection, instigating a complete system removal in most cases [9]. In different studies, the prevalence of CIED infection ranges from 0.5% to 4.8% with a peak observable one year after pocket manipulation [15,16]. The vast majority of CIED infections are caused by the normal skin flora, and local contamination is believed to be the typical mechanism of infection [13]. There are several well-known patient-related risk factors of CIED infection, such as diabetes mellitus, renal failure, chronic obstructive pulmonary disease, corticosteroid therapy, previous device infection, malignancy, heart failure, pre-procedural fever, anticoagulant therapy and skin disorders [13,17,18,19,20,21]. Classical device-related risk factors of CIED infection are the abdominal generator pocket, device type, presence of multiple leads, dual-chamber system and epicardial leads. Procedure-related risk factors include the duration of the procedure, hematoma formation, temporary pacing, inexperienced operator, lack of antibiotic prophylaxis, number of previous procedures, device upgrade, device replacement, device revision and lead repositioning [13,17,18,20,21].

In a prospective study, Ghani et al. showed that the most common indication (66%) for lead-related reintervention, within the first year after CIED implantation, was lead dislodgement with the right atrial and ICD leads being associated with the highest risk of dislocation [22]. In a study by Prutkin et al. [23], lead dislodgement was the second most common indication for early reintervention (after pocket hematoma), being associated with a significant risk for infection. In other studies, revision procedures were defined as lead or generator revision [21,24,25,26], without specific data regarding the revised lead type. In our study, we could not calculate the exact rate of lead dislodgement after primary CIED implantation. Regarding the frequency of lead dislodgement, Qin D et al. [27] reported a 0.95% rate in a large real-world registry, 70% of these events occurring early, within 3 months from implantation. In this study, coronary sinus leads and right ventricular leads were the most common among the revision procedures, while coronary sinus and right atrial leads presented the highest need for reintervention due to lead dislodgement. 

In such cases, patients must undergo another procedure with all potential complications. The most serious consequence of the need for reintervention after CIED implantation is definitely the elevated risk of infection. Further adverse effects could be the delayed or repeated hospitalization [28], extra load of the operating room, more severe postoperative pain, increased psychological stress of the patient or prolonged skin healing. Acute lead dislodgement may be also associated with increased risk for in-hospital death, as observed in a large US registry of patients undergoing ICD implantation [29]. Therefore, it recommended to make every effort and give faithful attention to reduce the risk of a complication requiring reintervention [30,31].

### 4.3. Reasons Supporting Delayed or Early Reintervention

As described above, the early device reintervention is associated with a higher risk of CIED infection. Therefore, some operators delay the reintervention by weeks; however, there is a lack of evidence supporting this strategy and the optimal time point for reoperation has until now never been investigated. In a large, prospective study, Klug et al. showed that early reintervention (defined as early when occurring before hospital discharge) in the case of lead dislodgement is linked to an elevated risk of infection although there were no data in this study about delayed reoperation [32]. The main argument behind the postponement of lead repositioning was a hypothetical reduction in the infection risk. In addition, the reduced effect of local anesthetics and the difficulty of operating on oedematose, inflamed tissue may also play a role.

On the other hand, a long lead dwell time is associated with the formation of encapsulating scar tissue around the intravascular leads and is therefore considered the most important risk factor of TLE [33]. Although a few weeks of postponement does not usually cause any relevant difficulty during lead repositioning, the early scar formation may negatively affect the local immune response.

On the contrary, dysfunctional or dislodged leads may require suboptimal device programming, like asynchronous single-chamber (i.e., VVI) instead of AV-sequential dual-chamber pacing (DDD), or result in safety concerns due to the low R-wave amplitude in an ICD system [34,35]. Moreover, sudden cardiac death secondary to ICD lead dislodgement was also reported [36]. In general, it seems to be most reassuring for both patients and attending physicians to discharge patients after the resolution of all potential complications and with a completely functioning CIED system. Nonetheless, our study showed no benefit of delaying reintervention over one week regarding CIED infection; moreover, there was a trend for better outcomes in the patients receiving an early intervention.

### 4.4. Treatment Options for Early Wound Infection

Although most CIED infections require complete system removal, in some selected cases extraction can be omitted. As it is stated by the EHRA consensus document, a superficial incisional infection should be differentiated from a pocket infection, as it involves only the skin and the subcutaneous tissue and hence does not require CIED system extraction [9]. In our study cohort, two out of the nine patients could be treated conservatively with close monitoring and prescription of oral antibiotics and developed no signs of CIED-associated endocarditis during the follow-up.

### 4.5. Limitations

Our study has several limitations, firstly being a retrospective study with a potential selection bias. We tried to eliminate or at least minimize these by collecting objective data and performing a multivariate statistical adjustment for the typical risk factors of CIED infection. The results of this study may be also biased by the exclusion of patients at high risk (i.e., patients with pocket hematomas or undergoing upgrade procedures). Another limitation is that not all laboratory markers and clinical data were available for all patients. Long-term follow-up data were also not collected. It should be also noted that the strict 1-week cut-off used in the current study for defining an early reintervention may have affected the study outcomes. Moreover, it poses a challenge to compare results with the majority of registries from the literature, where various definitions of early reintervention, such as before hospital discharge and 30 days; 6 weeks; and 3, 6 or 12 months post-implantation have been specified in reporting post-implantation events. Specific data regarding the experience of the operators was not available and therefore no statistical adjustment was possible for this well-known confounding factor. Lastly, due to the rare study endpoint, the study may be statistically underpowered.

## 5. Conclusions

In this bicentric study, delayed reintervention in patients with lead dislodgment after primary CIED implantation did not reduce the risk of CIED infection compared to patients undergoing an early (<1 week) reoperation. Moreover, there was a trend toward better outcomes in patients receiving an early intervention. While acknowledging the limitations of the present dataset, characterized by a low incidence of events and its derivation from real-world clinical practice rather than a randomized study design, an early lead revision during the initial hospitalization without any delay may be considered in the case of acute lead dislodgement in patients with de novo CIED implantations.

## Figures and Tables

**Table 1:** Baseline characteristics.

	Total	Early Revision (≤1 Week)	Delayed Revision (>1 Week)	*p*-Value
Number of patients	249	85	164	
Age in years (median (IQR))	72.0 (15.4)	76.0 (68.9–81.8)	69.4 (62.3–77.9)	0.001
				
Male sex	134 (54%)	50 (59%)	84 (51%)	0.254
Type of device				
VVI PM	48 (19%)	24 (28%)	24 (15%)	0.021
VVI ICD	26 (10%)	8 (9%)	18 (11%)	
DDD PM	105 (42%)	40 (47%)	65 (40%)	
DDD ICD	16 (6%)	4 (5%)	12 (7%)	
CRT-P	13 (5%)	1 (1%)	12 (7%)	
CRT-D	41 (17%)	9 (11%)	32 (20%)	
Number of leads				0.006
(mean ± SD)	1.9 ± 0.7	1.7 ± 0.7	2.0 ± 0.7	
(median (IQR))	2.0 (1)	2.0 (1–2)	2.0 (1–3)	
Diabetes	65 (26%)	22 (26%)	43 (26%)	0.954
Heart failure	76 (31%)	21 (25%)	55 (34%)	0.151
Fever prior to implant	2 (0.8%)	0 (0%)	2 (1.2%)	0.307
Anticoagulation	89 (36%)	29 (34%)	60 (37%)	0.700
NOAC full dose	19 (8%)	4 (5%)	15 (9%)	0.468
NOAC reduced dose	6 (2%)	2 (2%)	4 (2%)	
VKA	53 (21%)	17 (20%)	36 (22%)	
LMWH	11 (4%)	6 (7%)	5 (3%)	
Platelet inhibition	118 (47%)	43 (50%)	75 (46%)	0.467
Prolonged antibiotic therapy	17 (7%)	4 (5%)	13 (8%)	0.339
Corticosteroids	2 (0.8%)	1 (1%)	1 (0.6%)	0.635
Temporary pacemaker	28 (11%)	13 (15%)	15 (9%)	0.145
Creatinine in umol/L (median (IQR)) ^a^	93.5 (40.5)	97.2 (81–137.6)	89.0 (71–110.5)	0.023
White blood cell (WBC) count in/L) (median (IQR))				
At baseline ^b^	7.7 (3.2)	8.2 (6.6–10.4)	7.6 (6.4–9.2)	0.455
At revision ^c^	7.6 (2.8)	7.8 (6.9–10.4)	7.5 (6.3–8.9)	0.142
C-reactive protein (CRP) in mg/dL (median (IQR))				
At baseline ^d^	1.3 (6.3)	2.6 (0.4–12.5)	0.82 (0.2–4.1)	0.023
At revision ^e^	1.8 (9.0)	2.9 (0.9–14.4)	1.2 (0.6–7.8)	0.160

(^a^) Available for 216 patients. (^b^) Available for 187 patients. (^c^) Available for 179 patients. (^d^) Available for 96 patients. (^e^) Available for 94 patients.

**Table 2:** Clinical outcomes.

	Total	Early Revision (≤1 Week)	Delayed Revision (>1 Week)	OR, 95% CI, *p*-Value	Adjusted OR, 95% CI, *p*-Value
Number of patients	249	85	164		
Infection	9 (3.6%)	1 (1.2%)	8 (4.9%)	0.232 0.029–1.888 *p* = 0.172	0.264 0.032–2.179 *p* = 0.216
Explantation/extraction due to infection	7 (2.8%)	0 (0%)	7 (4.3%)	0.128 0.01–2.273 *p* = 0.161	N/A

CI = confidence interval, OR = odds ratio, and N/A = not applicable.

**Table 3:** Results of the univariate logistic regression analysis.

	OR	95% CI	*p*-Value
Early revision	0.232	0.029–1.888	0.172
Number of leads	0.790	0.310–2.013	0.621
Diabetes	1.435	0.348–5.913	0.617
Heart failure	1.144	0.278–4.699	0.852
Fever prior implant	29.875	1.711–521.624	0.020
Corticosteroid		N/A	
Anticoagulation	1.459	0.382–5.578	0.581
Platelet inhibition	0.543	0.133–2.224	0.396
Temporary pacemaker	0.986	0.119–8.191	0.990

CI = confidence interval, OR = odds ratio, and N/A = not applicable.

**Table 4:** Results of the multivariate logistic regression analysis (method: backward stepwise Wald).

	Adjusted OR	95% CI	*p*-Value
Step 1			
Early revision	0.264	0.032–2.179	0.216
Fever prior implant	22.143	1.251–391.868	0.035
Step 2			
Fever prior implant	29.875	1.711–521.624	0.020

CI = confidence interval, OR = odds ratio.

## Data Availability

All data used in this study are available by request from the corresponding authors.
